# Qualitative and quantitative comparisons of type 1 macular neovascularizations between pachychoroid neovasculopathy and neovascular age-related macular degeneration using optical coherence tomography angiography

**DOI:** 10.1038/s41433-024-03007-2

**Published:** 2024-03-12

**Authors:** Özge Yanık, Sibel Demirel, Gökçen Özcan, Figen Batıoğlu, Emin Özmert

**Affiliations:** 1 Department of Ophthalmology, Ankara University School of Medicine, Ankara, Turkey; 2 Polatlı Duatepe State Hospital, Ankara, Türkiye; 3 Bio-Retina Eye Clinic, Ankara, Turkey

**Keywords:** Retinal diseases, Macular degeneration

## Abstract

**Objectives:**

To compare qualitative and quantitative features of type 1 macular neovascularizations (MNV) in pachychoroid neovasculopathy (PNV) and neovascular age-related macular degeneration (nAMD).

**Methods:**

Forty-three treatment-naive eyes of 41 PNV patients and 40 treatment-naive eyes of 38 patients with nAMD were included. The patients were classified as PNV or nAMD according to the presence of pachychoroid features and soft/reticular drusen. Presence of central trunk and maturity of the MNV were evaluated on optical coherence tomography angiography (OCTA) images. MNV area, vessel density (VD), total vessel length (VL), number of intersection points (IPs), fractal dimension (FD), and lacunarity (LAC) were calculated using ImageJ software and FracLac plugin.

**Results:**

The mean age was 56.8 ± 8.7 years in PNV and 70.4 ± 8.8 years in neovascular AMD groups (*p* < 0.001). Compared to nAMD, the presence of central trunk was less frequent in PNV (48.8% vs 77.5%, *p* = 0.007). Immature MNV pattern was observed more frequently in PNV eyes than nAMD (41.9% vs 20.0%, *p* = 0.009). PNV cases had significantly lower median MNV area [0.913(1.115) vs 2.542(3.273) mm²], total VL [14.84 (20.46) vs 36.34 (44.68) mm], number of IPs [104(140) vs 335(417.3)], and FD [1.56(0.10) vs 1.59(0.11)] comparing to nAMD cases (*p* < 0.001, *p* = 0.001, *p* < 0.001, *p* = 0.043 respectively). However, the mean VD (42.4 ± 6.8 vs 42.9 ± 9.0%) and the median LAC values [0.42 (0.09) vs 0.42 (0.09)] did not differ significantly between groups (*p* = 0.776, *p* = 0.526, respectively).

**Conclusion:**

Morphological and quantitative differences exist in type 1 neovascular lesions. Type 1 MNVs in the PNV group are characterized by a smaller and less complex structure.

## Introduction

Macular neovascularization (MNV) is one of the visually devastating conditions and defined as an invasion of neovascular tissue into the outer retina, subretinal space, or subretinal pigment epithelial (sub-RPE) space. Type 1 MNV, previously known as occult choroidal neovascularization, means the ingrowth of vessels from the choriocapillaris into and within the sub-RPE space leading to varying types of pigment epithelial detachments (PEDs) [1].

Type 1 MNVs may occur in several retinal pathologies including neovascular age-related macular degeneration (nAMD) and pachychoroid spectrum diseases. Pachychoroid neovasculopathy (PNV) was first described as an MNV in the presence of thick choroid and absence of AMD or other degenerative pathologies in 2015 [2].

Differential diagnosis of a type 1 MNV in PNV and nAMD sometimes becomes challenging [3, 4]. Although the age of onset in PNV is relatively earlier, PNV and nAMD may occur in similar age groups [5, 6]. In some cases with PNV, the increase in choroidal thickness may be focal without affecting entire choroid [7]. Therefore, localized increase in choroidal thickness may obscure the recognition of pachychoroid features. The overlap of some imaging features may further complicate the differential diagnosis. For instance, the presence of dilated vessels may not be an exclusive characteristic unique to PNV. Baek et al. compared choroidal vascular characteristics of AMD, polypoidal choroidal vasculopathy (PCV), and central serous chorioretinopathy (CSC) cases, and reported the presence of pachyvessels even in non-neovascular (25%) and neovascular AMD (46%) groups [8]. In this case, the presence of typical drusen may be helpful in diagnosis, but typical drusen may not be seen around a large neovascular lesion or in the fellow eyes of cases with nAMD [9]. Such conditions pose difficulties in the differential diagnosis between these two diseases. Some cases previously diagnosed as AMD have been reported to be re-diagnosed as PNV [3, 4]. A study from Japan used machine learning to re-evaluate cases with nAMD and reported that 46% of cases had pachychoroid-related features and were reclassified as PNV [4]. This rate was quite striking in terms of showing the difficulty in the distinctive diagnosis of these two diseases. They also evaluated clinical outcomes and observed that the presence of pachychoroid-related features was significantly associated with a higher visual acuity improvement [4]. This finding was also important in terms of revealing the differences in the prognosis, further emphasizing the importance of differential diagnosis.

The number of studies comparing the properties of type 1 MNVs in these two groups is very limited in the current literature [5, 6, 10, 11]. Early studies used dye angiography for the comparison of neovascular networks [6, 11, 12]. Greatest linear dimensions of MNVs on dye angiography was reported to not differ in PNV and nAMD [10, 12, 13]. However, conventional dye angiographies have some limitations for quantitative measurements of membrane structures due to being affected by leakage, staining, and choroidal hyperpermeability.

Optical coherence tomography angiography (OCTA) allows detailed visualization of neovascular networks with their exact boundaries and intrinsic details without being affected by leakage, staining, and choroidal vascular hyperpermeability. Previous OCTA studies evaluating the features of type 1 MNVs in PNV and nAMD mainly focused on morphological analyses of the networks and some quantitative features limited to basic parameters such as the area or VD of the neovascular networks which could be directly measured by the software of the device [5, 10].

Quantitative evaluation of distinct features of type 1 MNV lesions in nAMD and PNV may provide a better understanding of the underlying pathophysiological mechanisms causing MNV formation in these disorders. The aim of this study was to compare qualitative and quantitative features of type 1 MNVs including morphological characteristics as well as MNV area, vessel density (VD), total vessel length (VL), number of intersection points (IP), fractal dimension (FD), and lacunarity (LAC) between PNV and nAMD cases to find an answer to the following question: Is the structure of type 1 MNVs similar regardless of etiology? If not, could a quantitative feature be identified that may be used in the differential diagnosis?

## Methods

Forty-three treatment-naive eyes of 41 PNV patients and 40 treatment-naive eyes of 38 patients with nAMD were included. This study was conducted in accordance with the Declaration of Helsinki. The institutional Review Board of Ankara University School of Medicine approved the study (27 April 2022 İ05-251-22). The medical records of patients who visited Ankara University School of Medicine from January 2016 to February 2022 were reviewed.

The diagnosis of type 1 MNV was based on both clinical examination findings and multimodal imaging modalities. Type 1 MNV was defined as ingrowth of vessels initially from the choriocapillaris into and within the sub-RPE space [1]. On spectral domain optical coherence tomography (SD-OCT), all patients had PEDs with signs of active neovascularization including subretinal and/or intraretinal fluid.

The diagnostic criteria for nAMD were based on Consensus Nomenclature for Reporting Neovascular Age-Related Macular Degeneration Data [1]. Individuals aged 50 or older presenting with Type 1 MNV with notable features including the accumulation of extracellular deposits such as subretinal drusenoid deposits, basal linear, and basal laminar deposits were included [1].

PNV was defined as type 1 MNV overlying pachychoroid disease changes appearing as shallow irregular PED (double-layer sign) on SD-OCT and “tangled network” of flow signal on OCTA in the absence of other identifiable risk factors for MNV such as soft drusen, myopic degeneration, inflammation, or angioid streaks [14].

Pachychoroid disease changes included [14]:

1. Focal or diffuse choroidal thickening on EDI-OCT: Subfoveal choroid may be normal but extrafoveal area of increase thickness (>50 µm more than subfoveal measurement) may be detected.
2. Dilated choroidal vessels (pachyvessels) on EDI-OCT: Increased diameter of choroidal vessel lumen on cross-sectional OCT. Large caliber vessels in Haller’s layer on en face OCT.
3. Thinning/absence of choriocapillaris and Sattler’s layer overlying pachyvessels on EDI-OCT.
4. Dilated choroidal vessels, choroidal filling defects, choroidal vascular hyperpermeability in mid to late phase on ICGA.

Due to the absence of a clear-cut distinction between PNV secondary to pachychoroid pigment epitheliopathy or de novo PNV and the neovascularization that complicates the disease, such as CSC, we included both cases: Eyes with PNV but no history or sign of a previous CSC attack, and eyes with PNV with a known previous CSC period. Fellow eye characteristics including choroidal hyperpermeability and the presence or absence of drusen were also considered.

Exclusion criteria were the presence of any other chorioretinal and/or inflammatory ocular diseases, aneurysmatic type 1 MNV, any evidence of type 2 or 3 MNVs, relevant opacities of the optic media preventing adequate imaging, a high refractive error ≥5.00 diopter spherical equivalent, low-quality OCTA images below 6/10 scan quality and/or with significant artifact, a history of previous photodynamic therapy or anti-vascular endothelial growth factor injection. Patients with any history of intraocular surgery within the past 6 months were excluded.

The subjects’ medical charts were reviewed for demographic and clinical data. All subjects underwent a comprehensive ophthalmic examination including best corrected visual acuity (BCVA) measurement using the ETDRS charts, intraocular pressure measurement, slit lamp biomicroscopy, and a dilated fundus examination. SD-OCT (Spectralis, Heidelberg Engineering Inc., Heidelberg, Germany) and OCTA (Avanti RT Vue XR® with AngioVue® software; Optovue Inc., Fremont, USA) were performed in all patients. BCVA scores were converted into the logarithm of the minimum angle of resolution (logMAR) values for statistical analysis.

Maximum height and maximum area of the PED on SD-OCT were measured manually using the calliper tool of the Heidelberg Eye Examination software. Maximum PED height was defined as the maximum vertical distance between from the RPE to Bruch’s membrane and measured using manual calliper tool. PED area was defined as the greatest PED area between Bruch membrane and the outer boundary of the RPE on the horizontal B-scan OCT and measured using manual calliper tool at the most prominent PED lesion site within the central 6-mm-diameter circle of the ETDRS grid.

A 6 × 6 mm volumetric cube of a 70,000 Hz (840-nm wavelength) OCTA system was used for the visualization of the MNV and morphological and quantitative analyses. The choriocapillaris slab of OCTA was automatically selected [15]. The thickness between the two segmentation lines was also manually adjusted to include the whole MNV complex [16, 17].

**Fig. 1:**
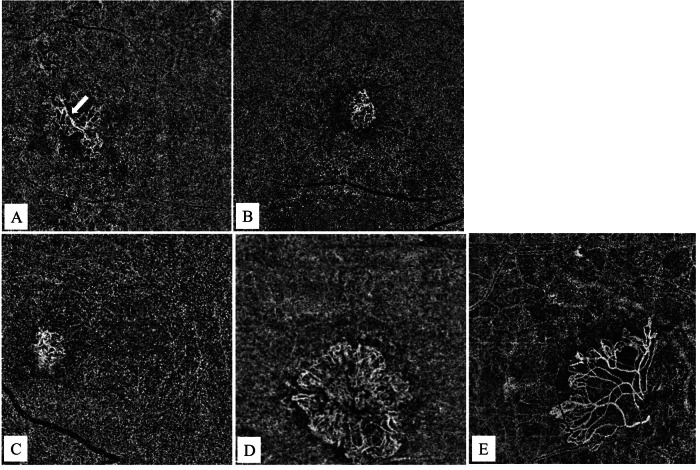
Qualitative morphological classification of macular neovascularizations (MNVs). Identification of the central trunk of a type 1 macular neovascularization (MNV) on the manually adjusted choriocapillaris slabs of en-face optical coherence tomography angiography: **A** A type 1 MNV with a visible central trunk (arrow) that is a single large vessel located anywhere within the lesion branching into smaller vessels. **B** A type 1 MNV without a detectable central trunk. Classification of maturity of type 1 macular neovascularization (MNV) according to the density of the capillaries on the manually adjusted choriocapillaris slabs of en-face optical coherence tomography angiography: **C** An immature type 1 MNV in the presence of a dense network of capillary vessels. **D** A mature type 1 MNV with reduced density of capillaries within a well-developed network of vessels. **E** A hyper-mature type 1 MNV which is composed of well-delineated vessels with almost no capillaries.

On en-face OCTA images, the morphologic patterns of MNV complexes were categorized according to the most recent recommendations of the International Panel of Experts on OCT Angiography Nomenclature of nAMD by UNICORN (UNIfied COmmentary of the committee of inteRnational experts on the nomenclature for Neovascular AMD in OCTA) group (Fig. 1) [18]. Central trunk was defined as a single large vessel located anywhere within the lesion branching into smaller vessels [18]. Maturity of the MNV lesion was categorized as immature, mature, and hypermature according to the density of the capillaries [18]:

Immature MNV: Dense net of capillary vessels.

Mature MNV: Reduced density of capillaries within a well-developed network of vessels.

Hyper-mature MNV: Almost no capillaries with net composed of well delineated vessels.

Two retina experts having experience more than 10 years (S.D., F.B.) blindly evaluated the OCTA images. In the case of conflict, a consensus was obtained by open adjudication. Cohen’s Kappa coefficients to quantify the intergrader agreement for qualitative findings were calculated.

**Fig. 2:**
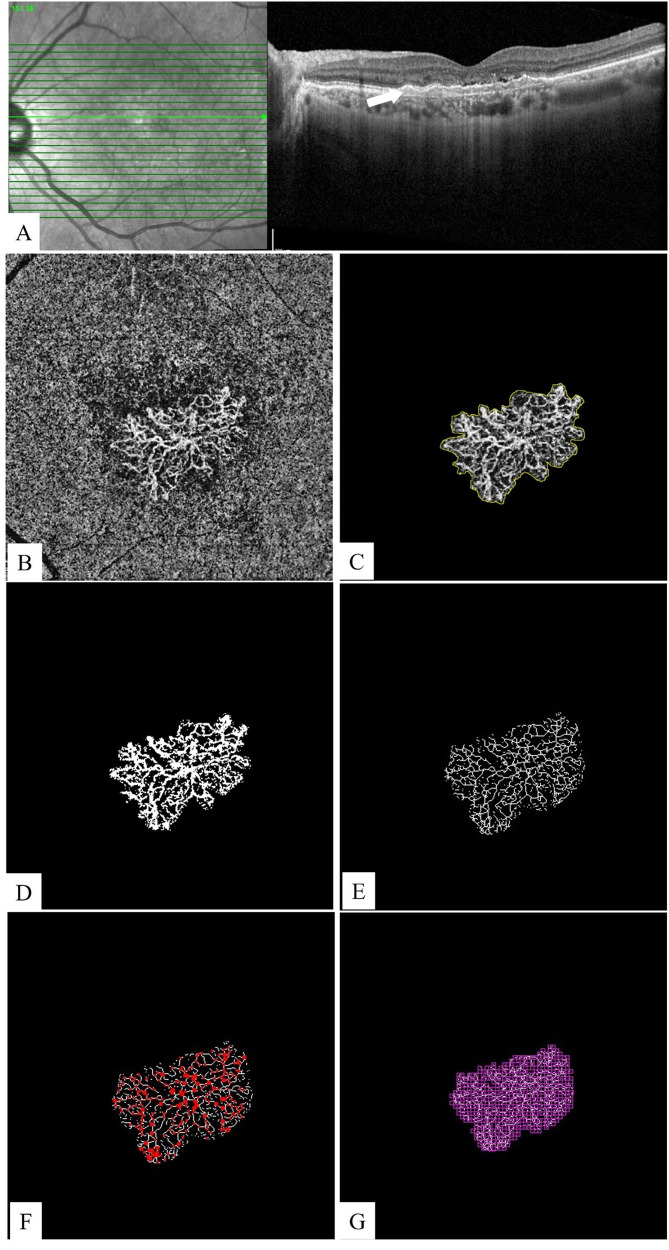
A 63-year-old patient with neovascular age-related macular degeneration. **A** A horizontal B-scan spectral domain optical coherence tomography image shows shallow irregular pigment epithelium detachment (arrow) with subretinal fluid. **B** A manually adjusted choriocapillaris slab of en-face optical coherence tomography angiography reveals type 1 macular neovascularization (MNV). **C** In ImageJ software, the area of the MNV was manually outlined and measured as 3.753 mm^2^. **D** Otsu binarization: The vessel density of the lesion was calculated as 42.99% which represents the ratio of the area occupied by vessels to the total area of the MNV. **E** Then the lesion was skeletonized, and the total vessel length of the lesion was measured as 52.779 mm using the Measure Skeleton Length Tool. **F** Skeleton intersection points were calculated as 476 by the skeleton analysis plugin. **G** To quantify morphological complexity and structural nonuniformity, the FracLac plugin of ImageJ software was used. The fractal dimension and lacunarity values were 1.653 and 0.39, respectively.

**Fig. 3:**
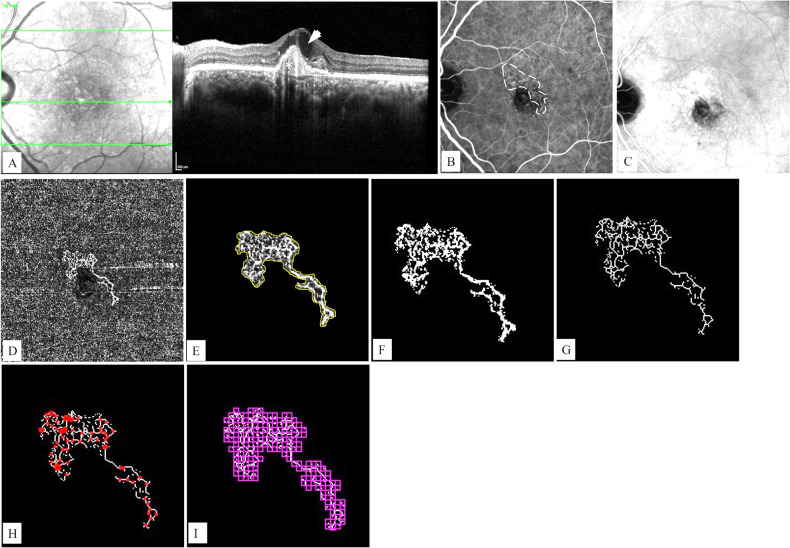
A 64-year-old patient with pachychoroid neovasculopathy. **A** A horizontal B-scan spectral domain optical coherence tomography image shows a peaked pigment epithelium detachment (asterisk), intraretinal cysts (arrow head), and increased subfoveal choroidal thickness with dilated outer choroidal vessels. **B** An early phase indocyanine angiography (ICGA) image shows a hypercyanescent fine neovascular network of type 1 macular neovascularization (MNV) (dotted outline). **C** Late phase of ICGA reveals a hypercyanescent plaque appearance and choroidal hyperpermability. **D** The manually adjusted choriocapillaris slab of en-face optical coherence tomography angiography reveals type 1 MNV. **E** In ImageJ software, the area of the MNV was manually outlined and measured as 1.182 mm^2^. **F** Otsu binarization: The vessel density of the lesion was calculated as 33.66% which represents the ratio of the area occupied by vessels to the total area of the MNV. **G** Then the lesion was skeletonized, and the total vessel length of the lesion was measured as 13.723 mm using the Measure Skeleton Length Tool. **H** Skeleton intersection points were calculated as 77 by the skeleton analysis plugin. **I** To quantify morphological complexity and structural nonuniformity, the FracLac plugin of ImageJ software was used. The fractal dimension and lacunarity values were 1.401 and 0.66, respectively.

Quantitative OCTA parameters, including MNV area, VD, VL, number of IPs, FD, and LAC were calculated using ImageJ program version 1.52 u bundled with 64-bit Java 1.80_112 (Wayne Rasband, National Institutes of Health, Bethesda, Maryland, USA, https://imagej.nih.gov/ij) and FracLac plugin (Figs. 2 and 3). The area of the MNV was manually outlined in ImageJ using Freehand Selection tools, which were then registered with the region of interest manager. The outside of the MNV was cleared. Otsu method was used then to binarize the OCTA image [16, 17, 19]. This method determines a threshold value that minimizes the variance in grayscale values within each class and maximizes variance between the classes [20]. The VD of the lesion was calculated as a ratio of the area occupied by vessels to the total area of the MNV. Then the lesion was skeletonized, and the total VL of the lesion was measured with the Measure Skeleton Length Tool. Skeleton IPs were calculated by the skeleton analysis plugin. To quantify vessel complexity, the FracLac plugin of ImageJ software was used with box-counting method. The box-counting method consists of dividing a vascular network into square boxes of equal sizes and counting the number of boxes containing a vessel segment. The logarithm of the box size is plotted against the number of boxes. Lacunarity from box counting is calculated as the coefficient of variation in pixel density. FD represents the morphologic complexity of the vascular structure, whereas LAC represents nonuniformity of the lesions [21]. Lesions with more heterogeneous structures show higher LAC values, while those with homogeneous vascular structures have lower LAC values [16].

The primary outcome measures were FD and LAC calculations of neovascular lesions to observe morphological complexity and structural nonuniformity in both groups. The secondary outcome measures were total VL and number of IPs as well as morphologic patterns of MNV complexes.

### Statistical analysis

The statistical analyses were performed using Statistical Package for Social Sciences; version 15.0. The variables were investigated using visual (histograms, probability plots) and analytic methods (Shapiro–Wilk test) to test the distribution of the data. Continuous variables were expressed as the mean ± standard deviation (SD) or median (interquartile range) depending on the normality. Categorical variables were expressed as the number of observations and/or percentages. For the comparison of ordinal categorical data (maturity of MNV), the Mann–Whitney U test was performed. Depending on the distribution of the data, the independent sample T-test or the Mann–Whitney U test was used for the comparisons of continuous variables. Cohen’s Kappa coefficient was calculated to quantify the intergrader agreement for qualitative variables. The level of significance was defined at *p* < 0.05.

## Results

Forty-three treatment-naive eyes of 41 PNV patients (21 females, 20 males) and 40 treatment-naive eyes of 38 patients (19 females, 19 males) with nAMD were included. Detailed demographic and clinical characteristics of patients are shown in Table 1.

**Table 1:** Demographic characteristics and best corrected visual acuity scores of the groups.

Patient characteristic, *n* = 79			
Variables	PNV, *n* = 41 Mean ± SD/ Median (IQ)	nAMD, *n* = 38 Mean ± SD/ Median (IQ)	*P* value
Age (years)	56.8 ± 8.7	70.4 ± 8.8	***p*** < **0.001**^**a**^
Female/Male (number)	21/20	19/19	0.914^b^
BCVA (LogMAR)	0.30 (0.50)	0.40 (0.55)	0.298^c^

Bold values indicate statistically significant *p* values.

*nAMD* Neovascular age-related macular degeneration, *PNV* Pachychoroid neovasculopathy.

^a^Independent T Test.

^b^Chi-square.

^c^Mann–Whitney U.

Morphological and quantitative features of type 1 MNVs on OCT and OCTA are given in Table 2. PNV cases had significantly lower median maximum PED height [56 (59) vs 116 (83) µm, *p* < 0.001] and PED area [0.060 (0.06) vs 0.185 (0.25) mm², *p* < 0.001] than neovascular AMD cases on SD-OCT. Compared to nAMD, the presence of central trunk was less frequent in PNV (48.8% vs 77.5%, *p* = 0.007). Also, the immature MNV pattern was observed more frequently in PNV eyes than nAMD (41.9% vs 20.0%, *p* = 0.009). For these morphologic characteristics, Cohen’s kappa coefficients between graders were excellent (0.898 to 0.936).

**Table 2:** Morphological and quantitative features of type 1 macular neovascularizations on optical coherence tomography (OCT) and optical coherence tomography angiography (OCTA).

	PNV, *n* = 43	nAMD, *n* = 40	*p* value
OCT	Median (IQ)	Median (IQ)	
PED maximum height	56 (59)	116 (83)	**<0.001**^**a**^
PED area, mm^2^	0.060 (0.06)	0.185 (0.25)	**<0.001**^**a**^
**Morphological OCTA findings**	***n*** **(%)**	***n*** **(%)**	
Maturity of MNV			**0.009**^**a**^
• Immature	18 (41.9)	8 (20.0)	
• Mature	23 (53.5)	24 (60.0)	
• Hypermature	2 (4.7)	8 (20.0)	
Central trunk present	21 (48.8)	31 (77.5)	**0.007**^**b**^
**Quantitative OCTA findings**	**Mean** **±** **SD/Median (IQ)**	**Mean** **±** **SD/Median (IQ)**	
MNV area, mm^2^	0.913 (1.115)	2.542 (3.273)	**<0.001**^**a**^
Vessel density, %	42.4 ± 6.8	42.9 ± 9.0	0.776^c^
Total vessel length, mm	14.84 (20.46)	36.34 (44.68)	**0.001**^**a**^
Intersection points, number	104 (140)	335 (417.3)	**0.001**^**a**^
Fractal dimension	1.56 (0.10)	1.59 (0.11)	**0.043**^**a**^
Lacunarity	0.42 (0.09)	0.42 (0.09)	0.526^**a**^

Bold values indicate statistically significant *p* values.

*nAMD* Neovascular age-related macular degeneration, *PED* Pigment epithelial detachment, *PNV* Pachychoroid neovasculopathy

^a^Mann–Whitney U.

^b^Chi-square.

^c^Independent T Test.

Regarding quantitative OCTA measurements, PNV cases had significantly lower median MNV area [0.913 (1.115) vs 2.542 (3.273) mm²], total VL [14.84 (20.46) vs 36.34 (44.68) mm], number of IPs [104 (140) vs 335 (417.3)], and FD [1.56 (0.10) vs 1.59 (0.11)] comparing to nAMD cases (*p* < 0.001, *p* = 0.001, *p* < 0.001, *p* = 0.043 respectively). However, the mean VD (42.4 ± 6.8 vs 42.9 ± 9.0%) and the median LAC values [0.42 (0.09) vs 0.42 (0.09)] did not differ significantly (*p* = 0.776, *p* = 0.526, respectively).

## Discussion

This study compared the qualitative and quantitative features of the type 1 MNV lesions in PNV and nAMD. To the best of our knowledge, this is the first study quantitatively comparing the heterogeneity and complexity of the MNV lesions in these diagnostic groups. According to the results of our study, type 1 MNVs in the PNV group are morphologically smaller and immature lesions with less frequent visualization of the central trunk. Regarding quantitative parameters, except for VD and LAC, all quantitative parameters were lower in the PNV group.

Recent studies have reported several results in favor of the existence of different underlying mechanisms in MNV development between PNV and nAMD [6, 12]. In PNV, vortex vein stasis leads to compression of choriocapillaris, which in turn produces occlusion of the choriocapillaris causing chronic localized ischemia [22]. However, in AMD, extensive choriocapillaris loss was observed in even early and intermediate stages, and the attenuation of CC progresses with the severity [13].

Genetic background and cytokine profiles in cases with PNV and nAMD were also different [6, 11, 12, 23]. Regarding nAMD, clinical and genetic data support the association of inflammatory pathways in the pathogenesis of AMD [24]. Risk alleles in complement factor H (CFH) and age-related maculopathy susceptibility 2 (ARMS2) were reported to be associated with early and advanced AMD [24]. However, PNV cases were known to be less genetically susceptible to AMD [6]. Genotype distribution of AMD susceptibility single nucleotide polymorphisms (both ARMS2 A69S and CFH I62V) differed significantly between PNV and nAMD [6]. Furthermore, intraocular vascular endothelial growth factor (VEGF) concentrations was reported to be lower in PNV cases in contrast to nAMD [11, 12, 23]. As a result, the overload of angiogenetic cytokines and factors in nAMD may further increase lesion growth and that may be an explanation for why type 1 MNVs in nAMD were found to be larger in our study.

The first OCTA study evaluating the features of type 1 MNV in PNV compared with nAMD was conducted by Arf et al. [10]. They reported that most of the lesions had well-defined morphology and lesion size and flow area on en face OCTA images were similar in both groups. However, Biçer et al. demonstrated that type 1 MNVs in the PNV group had a smaller MNV area and lower flow characteristics compared to nAMD [5]. They also observed that the presence of a feeder vessel was more common in AMD than in PNV whereas the indistinct pattern was more common in PNV than AMD. Altınışık et al. also reported that the MNV area was smaller in PNV group (0.77 ± 0.54 mm^2^ vs 1.57 ± 1.43 mm^2^) but did not reach significant levels due to the small number of cases [25]. The results of our study are consistent with those of the previous study by Biçer et al., a central trunk was more frequently seen in nAMD and all quantitative parameters except for VD and LAC including MNV area, total VL, number of IPs, and FD were lower in the PNV group. We observed that PNV lesions were frequently immature with dense capillary networks, whereas nAMD membranes were commonly mature lesions with a well-developed network. It was previously shown that as the neovascular lesion grows, remodeling and enlargement of the feeding and draining vessels occur both in the choroid and within the lesion [26]. In our study, although both groups consisted of treatment-naïve cases, nAMD lesions were larger. Therefore, it can be speculated that a nAMD lesion, under the effect of higher VEGF concentrations, grows faster completing the maturation process and remodeling earlier than a PNV lesion. Supporting this hypothesis, the number of IPs was also high in nAMD because of the high number of vessel-branching points.

The recent studies in the evaluation of vascular lesions introduced newer concepts in the quantitative analysis of MNV lesions. The morphology of the MNV may also be assessed by its complexity and homogeneity. The FD is a measure of lesion complexity [21]. It is scored from 0 to 2: A high number indicates increased pattern complexity of the lesion. Lacunarity describes the distribution of the sizes of gaps or lacunae surrounding the object within the image and is a measure of vessel diversity. High values correspond to a heterogeneous, and low values to correspond a homogeneous vascular structure [16]. It has been reported that there is a correlation between the branching pattern of the lesion and the complexity expressed by the FD. The results of VD (42.9 ± 9.0%) and FD [1.59 (0.11)] measurements in nAMD were very similar to a previous study performed by Al-Sheikh et al. [27]. They reported a mean of 0.417 VD and 1.574 FD in treatment naïve active nAMD cases [27]. However, these FD values were lower than that reported by Serra et al. [17]. This discrepancy may be explained by the use of different imaging software to calculate FD values (Image J FracLac Plugin vs. Fractalyse vs. MATLAB coding language, respectively). Another important point that may affect the reliability of these measurements is that several OCTA devices have been shown to produce measurements with different mean values of MNV area, VD, and FD for the same cases [28]. The Bland-Altman analysis revealed these differences were not within the acceptable limits of agreement [28]. Therefore, the use of a single device, which is one of the strengths of our study, is important for the standardization of OCTA measurements for research and clinical practice. In our study, PNV group had lower FD values with less complexity, however nAMD cases had higher FD values with more complex structures. Since the cases in both groups were treatment-naïve, most of them had abundant capillary branching, and therefore, no difference was observed in LAC measurements.

Understanding the differences in type-1 MNVs secondary to either nAMD or pachychoroid diseases may provide valuable insights into their differential diagnosis. The differential diagnosis of these two diseases, which might affect similar age groups, is crucial for various reasons such as availability of the different treatment modalities such as photodynamic therapy [29, 30], variations in anti-VEGF therapy response [31], and differences in the prognosis of the diseases themselves [31]. Comparing to nAMD, Yoon et al. reported lower need for anti-VEGF injections and lower recurrence rate in PNV cases [31]. Moreover, PNV cases had a lower risk for macular atrophy development in contrast to nAMD [31]. Demirel et al. reported half-fluence PDT as an effective treatment alternative in PNV providing total resolution of subretinal fluid in 75.0% of the cases without any anti-VEGF injections [29].

The major limitations of the study were its small sample size, retrospective nature, and cross-sectional single-center design. Additionally, the use of manual segmentation method for the analysis of MNV, aiming to visualize all MNV, is one of the limitations. Although some OCTA software implementations are capable to reduce the projection, motion, and image processing artifacts of OCTA, there are still a significant number of images that could not be included due to their poor quality and/or artifacts. Due to the requirement for high-quality images for image analysis, the study may have been subject to selection bias. Moreover, venous insufficiency and hyperpermeability in pachychoroid diseases may theoretically lead to blood flow reduction in choriocapillaris due to venous stasis [22]. Therefore, it can be presumed that the flow in PNVs may also be slow causing a possible limitation of OCTA for MNV detection. Despite all these limitations of the study, we observed significant morphological and numerical differences between the two diagnostic groups proving that the nature of the type 1 MNV lesions in pachychoroid and nAMD differs. Future longitudinal studies are required to enlighten the developmental pathways, and to examine the effect of lesion characteristics on prognosis and the change of lesion properties over time.

In conclusion, the study showed morphological as well as quantitative differences in type 1 neovascular lesions between the two disorders. Type 1 MNVs in the PNV group are characterized by a smaller and less complex structure than in nAMD group. The distinct pathogenesis behind the formation of type 1 MNV lesions in these groups may explain these quantitative and qualitative differences. It may be possible to plan a more effective treatment strategy by being aware of these structural differences.

## Summary

### What was known before

- Type 1 MNV may occur in several retinal pathologies including nAMD and pachychoroid spectrum diseases.

### What this study adds

- This study revealed that type 1 MNVs in the PNV group are characterized by a smaller and less complex structure than in nAMD group. These differences in the morphology of these lesions revealed the distinct pathogenesis behind the formation of that type 1 MNVs in nAMD and PNV. For this reason, it is important to provide an accurate diagnosis of the etiology of MNV in these groups of patients with similar age, since the need for treatment may also differ.

## Data Availability

The datasets generated during and/or analyzed during the current study are available from the corresponding author on reasonable request.
